# Supplementary material for: Myocardial B cells have specific gene expression and predicted interactions in dilated cardiomyopathy and arrhythmogenic right ventricular cardiomyopathy
Source: Front Immunol. 2024 Apr 26;15:1327372. doi: 10.3389/fimmu.2024.1327372 (PMC11082303; doi:10.3389/fimmu.2024.1327372)
Supplement: Supplementary file 13 [file Image_1.pdf]

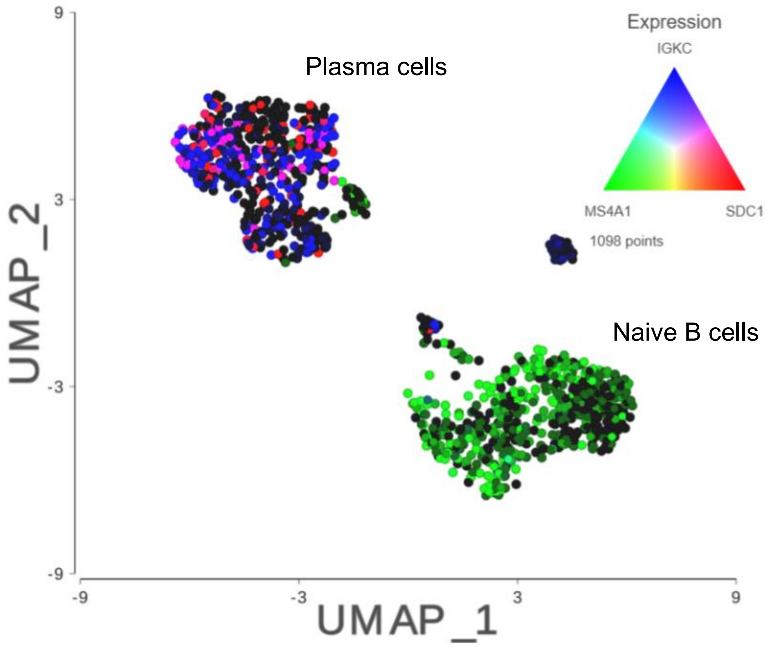

Supplementary Figure 1. UMAP of the integrated and classified B cells from all datasets showing the markers for naive B cells and plasma cells. B cells were subsetted from the integrated data and sub-classified into B-cell subtypes. The colors represent the expression levels of MS4A1 (CD20, green) highlighting the naive B cells; SDC1 (CD138, red); and IGKC (Ig Kappa Chain C, blue), highlighting the plasma cells. SDC1 was not detected in naïve B cells but it was present in plasma cells. Data were normalized, transformed, and scaled using SCTransform with 3000 variable features, and ScType was used for cell classification.

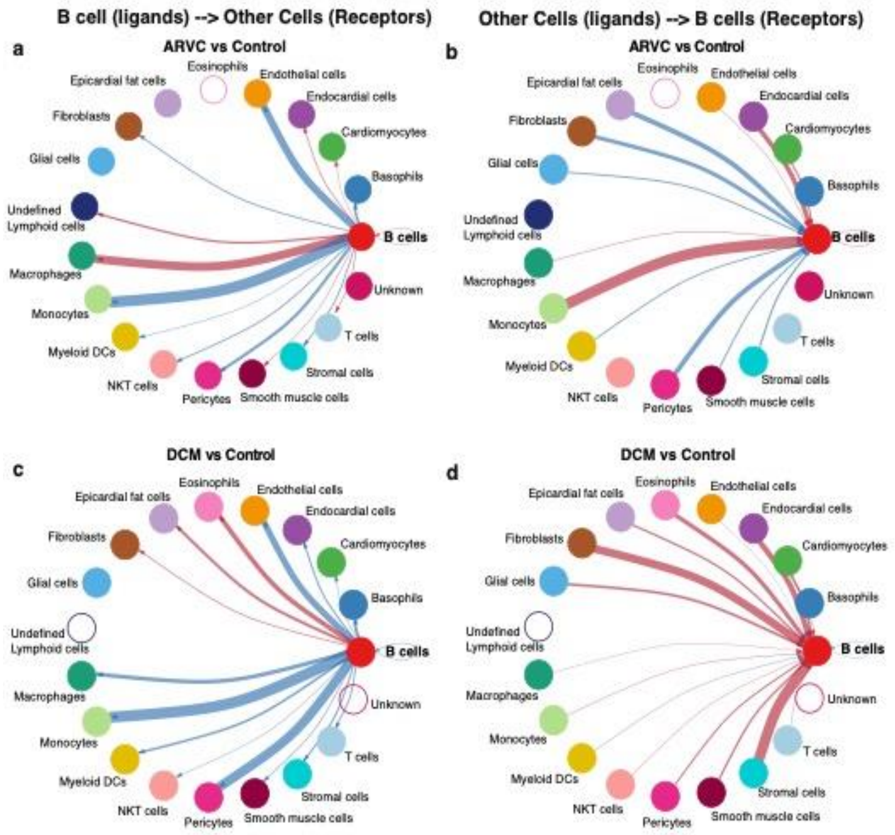

Supplementary Figure 2. Circle plot of B cell interaction as compared to control. The difference of interaction strength of B cell ligands to other cell's receptors between a) ARVC and controls and c) DCM and controls, and difference of interaction strength of other cell ligands to B cell receptors between b) ARVC and controls and d) DCM and controls are depicted. Thickness of the line is relative to the maximum difference in communication probability between disease conditions. Blue line color represents decreased interaction strength, red represents increased interaction strength. Open circles represent cell types that are not detected in either of the disease states being compared. DC = dendritic cell.

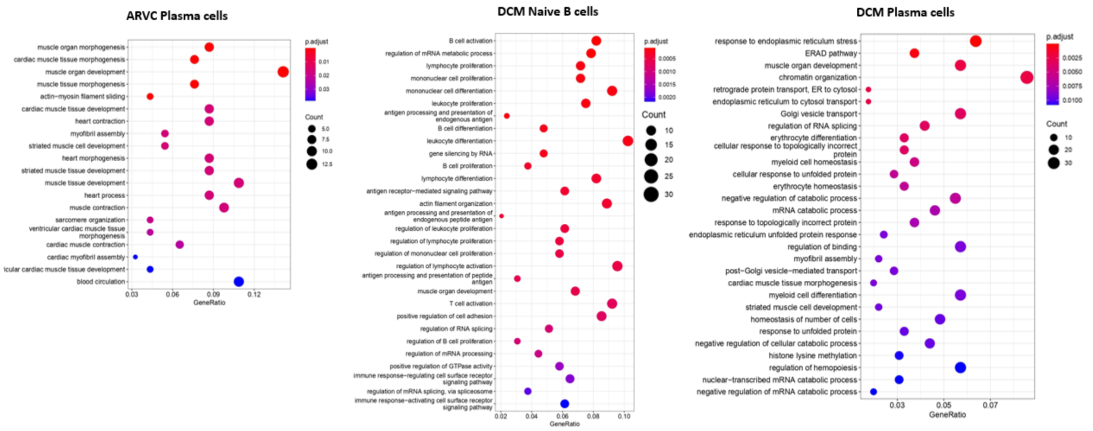

Supplementary Figure 3. Gene ontology enrichment analysis of Naive and Plasma B cells in DCM or ARVC cardiac samples vs control. a) Dysregulated gene ontology pathways in Plasma cells from ARVC samples. – No dysregulated gene ontology pathways were identified when comparing naive B cells from ARVC vs controls. b) Dysregulated gene ontology pathways in Naive B cells from DCM samples. c) Dysregulated gene ontology pathways in Plasma cells from DCM samples. DEGs with p-value < 0.05 calculated using DESeq2 and absolute fold change >1.5 were used for this analysis.
